# The immune microenvironment and tissue engineering strategies for spinal cord regeneration

**DOI:** 10.3389/fncel.2022.969002

**Published:** 2022-08-04

**Authors:** Yuan Feng, Yong Peng, Jing Jie, Yumin Yang, Pengxiang Yang

**Affiliations:** ^1^Key Laboratory of Neuroregeneration of Jiangsu and Ministry of Education, Co-innovation Center of Neuroregeneration, Nantong University, Nantong, China; ^2^Department of Clinical Laboratory, The First People’s Hospital of Nantong, The Second Affiliated Hospital of Nantong University, Nantong, China; ^3^Institute of Cancer Prevention and Treatment, Heilongjiang Academy of Medical Science, Harbin Medical University, Harbin, China

**Keywords:** spinal cord injury, immune microenvironment, regeneration, immune cells, biomaterials

## Abstract

Regeneration of neural tissue is limited following spinal cord injury (SCI). Successful regeneration of injured nerves requires the intrinsic regenerative capability of the neurons and a suitable microenvironment. However, the local microenvironment is damaged, including insufficient intraneural vascularization, prolonged immune responses, overactive immune responses, dysregulated bioenergetic metabolism and terminated bioelectrical conduction. Among them, the immune microenvironment formed by immune cells and cytokines plays a dual role in inflammation and regeneration. Few studies have focused on the role of the immune microenvironment in spinal cord regeneration. Here, we summarize those findings involving various immune cells (neutrophils, monocytes, microglia and T lymphocytes) after SCI. The pathological changes that occur in the local microenvironment and the function of immune cells are described. We also summarize and discuss the current strategies for treating SCI with tissue-engineered biomaterials from the perspective of the immune microenvironment.

## Introduction

Physical injuries of the spinal cord and neurodegenerative diseases often cause irreversible damage and loss of function, and as many as 3.6 to 195.4 cases per million people suffer from spinal cord injury (SCI) each year worldwide (Jazayeri et al., 2015; Kumar et al., 2018; Flack et al., 2022; Varadarajan et al., 2022). At present, SCI is a serious clinical problem that lacks effective treatment, especially secondary injury caused by the activation and infiltration of immune cells (Ahmed et al., 2018). The harsh microenvironment after SCI greatly hinders nerve regeneration and repair. In this process, the primary influencing microenvironmental cues are complex, including insufficient intraneural vascularization, prolonged immune responses, overactive immune responses, dysregulated bioenergetic metabolism and terminated bioelectrical conduction (Li et al., 2018a; Qian et al., 2021). Successful regeneration of injured nerves requires the intrinsic regenerative capability of the neurons and a suitable microenvironment (Park et al., 2008; Moore et al., 2009; Du et al., 2015). Nerve cells, glial cells, endothelial cells, fibroblasts and immune cells contribute to the formation of the local microenvironment. In addition to these cells, the extracellular matrix (ECM) and factors that produce cell-to-cell signals profoundly influence regeneration (Yang et al., 2021a). Efforts to promote injured nerve regeneration, particularly by removing unfavorable inflammatory factors, have been met with mixed success. The dual roles of immune cells within the inflammatory microenvironment are one key reason. Here, we discuss the structural characteristics of the spinal cord and significant clinical progress. A brief overview of the model systems that are most commonly used to study SCI is described. Importantly, we systematically summarize our current understanding of mammalian SCI responses to injury and highlight key advances in immune cells (neutrophils, macrophages, microglia, and T cells) in reconstructing the immune microenvironment. Moreover, we focus on the effects of biomaterials on SCI regeneration through regulating immune cells because these events have become the new direction of regenerative approaches.

### Spinal cord structure

**FIGURE 1:**
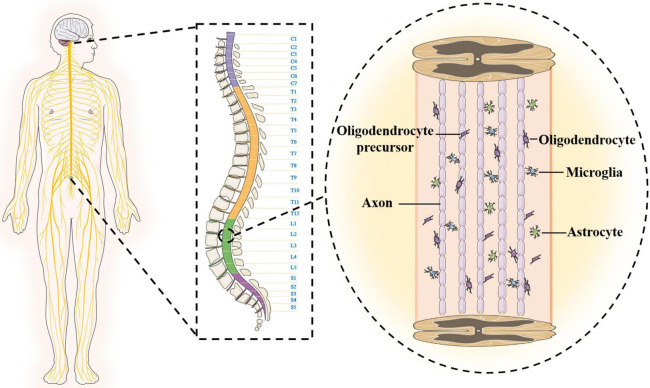
The structural characteristics of spinal cord.

The anatomical structure and cellular composition of the central nervous system are more complex than those of the peripheral nerves, which is one of the reasons why SCI is difficult to overcome. The spinal cord is located in the spinal canal, and the upper end joins the medulla oblongata at the foramen magnum (Shechter et al., 2013). The shape of the spinal cord is slightly flat, and there are two enlargements (cervical and lumbosacral). The spinal cord gradually tapers to form the conus medullaris below the lumbosacral enlargement and terminally forms the filament. The outermost layer of the surface of the spinal cord is wrapped by the hard spinal cord. The upper end is attached to the foramen magnum, and the lower end is attached to the coccyx. The middle layer is a translucent membrane encased by the spinal cord arachnoid. The innermost layer is the pia mater and is filled with cerebrospinal fluid. There is a large gap between the innermost layer and the arachnoid (Canaani et al., 2011; Chen et al., 2017). In addition, the surface of the spinal cord is not smooth and is composed of six longitudinal grooves or fissures. The spinal cord is mainly composed of neurons and glial cells, and the neuron cell body is mostly composed of dendrites, which aggregate to form gray matter. The central canal runs through the spinal cord, connects the fourth ventricle above, and reaches the conus medullaris below to form the terminal chamber, containing cerebrospinal fluid. In adults, the central canal is often regarded as vestigial, with studies showing that it is occluded and disassembled during the second decade of life (Garcia-Ovejero et al., 2015; Saker et al., 2016). The gray matter exhibits an “H” shape surrounding the intermediate canal, which consists of anterior horns, posterior horns and a median zone. White matter is loaded around gray matter and is mainly composed of nerve fibers, glial cells and blood vessels. Longitudinal fiber tracts in the white matter form the connection pathways between the brain and spinal cord. The glial cells are around neurons, and microglia are ependymal cells that line the central lumen of the spinal cord (Bradbury and Burnside, 2019; Courtine and Sofroniew, 2019). The oligodendrocyte initiates multiple processes that contact and wrap around the axon to form the myelin sheath. The soma of astrocytes sends out many long and branched protrusions, which stretch and fill between the soma and the protrusions of nerve cells to support and separate. The ends of astrocytes are attached to adjacent capillary walls (Figure 1).

### Clinical progress

The central nervous system of adult mammals has limited plasticity, and axons cannot regenerate spontaneously in the harsh microenvironment after severe spinal cord injury (Curcio and Bradke, 2018). At present, most preclinical strategies are only effective in animal models. There is no effective treatment to restore severely damaged spinal cord function. The clinical treatment of SCI is mainly aimed at the acute phase, including early immobilization of the damaged part of the spinal cord, palliative surgical decompression, vasopressors to increase mean arterial pressure (MAP) and selective injection of corticosteroids, such as methylprednisolone (Ahuja et al., 2017; Jutzeler et al., 2018; Samano and Nistri, 2019). It is worth noting that injection of methylprednisolone contributed little to the recovery of nerve function. These treatments are often conservative and do not greatly improve regeneration of the injured nervous system, and high-dose injections of methylprednisolone also increase the risk of adverse events. In addition to these treatments, drug therapy strategies targeting at alleviating secondary SCI have also emerged (Baroncini et al., 2021). Granulocyte-colony stimulating factor (G-CSF) is a glycoprotein that stimulates the bone marrow (BM) to produce granulocytes and stem cells, and it is mainly used in neutropenia. Moreover, G-CSF also has the neuroprotective effect of reducing cell inflammation and apoptosis. In phase I/II clinical trials, G-CSF was administered intravenously for 5 days in patients with acute SCI, and no serious adverse effects were observed (Koda et al., 2018). In a multicentre, prospective, non-randomized, non-blinded, comparative control study (phase IIb clinical trial), ASIA motor score was significantly improved in the G-CSF group (Inada et al., 2014). Minocycline, as an antibiotic, has properties of reducing inflammation and oxidative stress. Minocycline also exerts neuroprotective effects by inhibiting apoptosis. In a phase II clinical trial, the serum concentration of minocycline was stabilized for 7 days, and there were no obvious adverse reactions after SCI treatment. Furthermore, minocycline was proved to be feasible and safe in the above study, and associated with a trend of improvement in several outcome indicators (Casha et al., 2012). In addition, a variety of drugs can alleviate secondary SCI by reducing excitotoxicity and the inflammatory response, inhibiting apoptosis, promoting angiogenesis and/or supporting neuronal cells. These drugs include riluzole, basic fibroblast growth factor, glyburide, imatinib and AC-105 (Casha et al., 2012; Samano and Nistri, 2019; Takami et al., 2020; Baroncini et al., 2021; Flack et al., 2022). Most of these drugs have demonstrated efficacitive to some extent in animal studies and early clinical trials. Unfortunately, no drugs have been approved for the treatment of SCI (Koda et al., 2018; Takami et al., 2020).

### Stem cell treatment

Stem cell transplantation for SCI has significant advantages as follows: (1) Compared with the implantation of inert materials, it avoids the harm caused by the secondary surgical; (2) transplanted cells rapidly fill the defect of the lesion site; and (3) various nutritional factors are secreted and promote the regeneration of axons. Recently, stem cell transplantation has become the most promising preclinical treatment strategy for SCI (Bryukhovetskiy et al., 2005). Human embryonic stem cells (hESCs), induced pluripotent stem cells (iPSCs), ependymal stem/progenitor cells and mesenchymal stem cells (MSCs) have been used frequently in SCI research (Bryukhovetski et al., 2005; Okano, 2009; Blasko et al., 2017; Ning et al., 2019). hESCs have the ability to differentiate into ectodermal cells, such as neurons and glial cells. The transplantation of oligodendrocytes and neuronal progenitors derived from embryonic stem cells has achieved some success in animal models. iPSCs can differentiate into neural progenitor cells, neuronal cells and oligodendrocytes. The transplantation of iPSCs promotes functional recovery in the early stage of injury, but it has a risk of tumorigenicity (Wertheim et al., 2022). At present, stem cells are still controversial due to ethical issues, immune rejection and other issues. MSCs have significant advantages because they are isolated from BM, umbilical cord, fat and other tissues without causing harm to health or ethical controversy (Ma et al., 2018; Li L. et al., 2020; Zhu et al., 2021). The survival rate of MSCs can be improved by loading neurotrophic factors or cytokines with biological material scaffolds, such as hydrogels or ECM.

There are different applications of biological materials in support of stem cell transplantation to treat SCI. One way is to fill spinal cord defects with exogenous stem cells, which can differentiate into neurons and glial cells to promote spinal cord regeneration. Natural or artificial biological material scaffolds can directly load cells to repair SCI. Wang N. et al., 2018 used functional hydrogels or collagen-loaded MSCs to bridge defects of the spinal cord, which promoted axon growth and regeneration of neurons as well as significantly improved motor function recovery (Wang N. et al., 2018; Yin et al., 2018). The other way is to activate endogenous neural stem cells and induce their differentiation into functional neurons by scaffold loading neurotrophic factors, cytokines or small molecules (Fan et al., 2017; Yang et al., 2021d; Yin et al., 2021). This approach involves fewer ethical issues and is not limited to stem cell sources, stem cell numbers and low survival rates. Yang et al. (2015) used chitosan scaffolds loaded with neurotrophic factor-3 (NT-3) to provide an excellent microenvironment that activates endogenous neural stem cells at the injury site; the slow release of NT-3 promotes spinal cord nerve growth and functional recovery (Duan et al., 2015; Yang et al., 2015; Rao et al., 2018). The nervous injury microenvironment is not conducive to the survival of stem cells. Stem cell-derived exosomes are another way to achieve neural regeneration and protection. Exosomes are evenly dispersed in hydrogels, forming delivery systems that improve spinal cord regeneration in a more precise manner. Mu et al. (2021) coated human MSC-derived exosomes with a peptide-modified adhesive hydrogel to regulate the microenvironment of SCI and reduce inflammation and oxidative reactions, which is conducive to spinal cord regeneration and protection.

### Tissue engineering strategy

Approaches that focus on cell transplantation have failed to improve the complex, multilayered and spatiotemporal dynamics of the SCI microenvironment. The difficulty of transplantation and endogenous recruitment cells increases with high levels of reactive oxygen species or nitrogen (ROSN)accumulation under ischemia, excessive tissue remodeling under inflammatory response, excitability and cytotoxicity as well as glial scar formation (Bi et al., 2021). The combination strategy based on scaffolds can provide an excellent microenvironment for nerve regeneration, and it is mainly performed based on the two aspects of promoting growth or eliminating inhibitive factors (Fu et al., 2022). Various studies have been performed to promote spinal cord regeneration through scaffold load cells or nutritional factors. Natural biological materials (chitosan, collagen, gelatin, hyaluronic acid and cell matrix., etc.) or synthetic biodegradable biomaterials (polyglycolic acid, polylactic acid, polylactic acid-glycolic acid and polyepsilon-caprolactone., etc.) mimic the natural structure of the spinal cord for neuron proliferation, migration and differentiation (Chen et al., 2017; Varone et al., 2017; Shen et al., 2022a). Moreover, neurotrophic factor (NT-3), brain-derived neurotrophic factor (BDNF), basic fibroblast growth factor (bFGF) and vascular endothelial growth factor (VEGF) can be used alone or in combination to improve the survival of neurons and promote axonal regeneration (Li et al., 2009, 2019; He et al., 2011; Han et al., 2015; Chen et al., 2018; Wang X. L. et al., 2018; Shang et al., 2019).

During the acute period of SCI, many immune cells (macrophages, neutrophils, microglia and lymphocytes) are activated and infiltrate the lesion site. Severe inflammation and oxidation reactions hinder spinal cord regeneration, and some even persist for the rest of the patient’s life. Many researchers use hydrogels, nanoparticles and immunomodulatory scaffolds to deliver anti-inflammatory or antioxidant drugs or factors to treat SCI. These strategies improve the immune microenvironment of the damaged site and enhance regeneration capacity (White-Schenk et al., 2015; Dombrowski et al., 2017; Ham and Leipzig, 2018; Bi et al., 2021; Gao et al., 2021; Shen et al., 2022b). This review focuses on the pathological changes in the local microenvironment and the changes and functions of immune cells after SCI. The current strategies for treating SCI with tissue-engineered biomaterials are also discussed from the perspective of the immune microenvironment.

## Model for spinal cord injury regeneration

**FIGURE 2:**
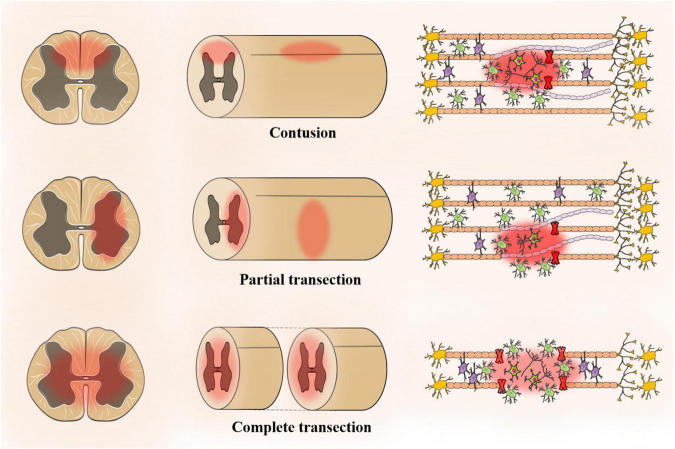
The animal model for spinal cord injury regeneration.

The animal model should be similar to human pathophysiology, morphological structure, electrophysiology and many other aspects. The ideal experimental animal model of SCI has the following characteristics: (1) ensure repeatability and reliability; (2) similar to anatomical structure and pathophysiology; (3) adjustable SCI degree; and (4) low cost, simple model and strong operability. The application of rats or mice remains optimal for preclinical studies of SCI. A review of 2,209 studies (excluding review articles and unoriginal articles) indicated that 92% of SCI models use rodents with 72.4% of the rodent models being rat models, which is attributed to rat models being easier to operate than mouse models (Sharif-Alhoseini et al., 2017). Moreover, rats are more similar to humans in terms of pathophysiology, morphological structure and electrophysiology. However, 16% of the models are mouse models, and the mouse genome is closer to that of humans. At present, SCI models include contusion, transection injury, compression injury, ischemia injury and photochemical injury models. Among them, the contusion model and transection injury model (partial and complete) account for a large proportion (Figure 2; Abdullahi et al., 2017).

### Contusion model

Allen (1991) first attempted to create a model of SCI that could be controlled by using an object to fix the height of the spinal cord. Since then, SCI models have undergone design improvements in terms of repeatability and quality control, including height, time and speed (Gruner, 1992). The most common impact method is weight loss (∼37.5% of all studies) followed by impact (∼27.4%) and infinite impact (∼20.6%) (Sharif-Alhoseini et al., 2017). In addition, there is also an air gun for SCI modeling in rats, which avoids lamina resection. However, its repeatability and stability need to be verified (Marcol et al., 2012).

### Transverse damage model

Allen (1991) showed that the injury progression of the contusion and transverse fracture have a similar trend in the acute phase within three weeks, but the pathophysiology and tissue morphology of the injury site are completely different (All and Al-Nashash, 2021). There are two types of transverse injuries, namely, partial and complete. In clinical practice, complete spinal cord transection is rare. Although complete spinal cord transection is not suitable for neuroprotective research, it is suitable for testing the performance of multifunctional biomaterial scaffolds, which explore neural regeneration, plasticity and tissue engineering strategies (Kim et al., 2018). The complete transverse injury model is suitable for cell transplantation. Lukovic et al. (2015) proposed a detailed and standard surgical procedure for a complete transect model and restored motor function in the transplant group to support the complete transect model for cell transplantation (All and Al-Nashash, 2021). After the lamina is surgically removed, the spinal cord is transected completely or partially using surgical scissors or blades (Talac et al., 2004). One side of the spinal cord is resected, while the spinal cord bundle is completely or partially preserved on the other side. The complete transect model facilitates the study of different functions of the damaged or non-damaged spinal tract. Neuroanatomic and electrophysiological studies have used a transverse injury model to assess the recovery of spinal motor function.

### Compression damage model

The majority of studies have used a spinal cord compression injury model with aneurysm clips (Sharif-Alhoseini et al., 2017). Rivlin and Tator (1978) first removed the rat spinal laminae and then molded them using a modified version of the aneurysm clip, and they quantified the relationship between the duration of compression injury and the severity of SCI by applying compression forces of 180 g or other to the spinal cord area for varying lengths of time. The advantages of the improved model are lower cost and relatively simple operation, but the actual compression force at the clipping site is difficult to accurately grasp (von Euler et al., 1997; Marques et al., 2014). Tarlov et al. (1953) inserted a balloon catheter into the spinal cord and then injected air or saline to compress and cause injury. Lim et al. (2007) measured the degree of spinal canal occlusion by injecting contrast agent into the catheter based on Tarlov’s method, and they also assessed the severity of SCI by HE.

### Ischemic injury model

Ischemia injury has been established in primates, large animals and rodents (Awad et al., 2021). It is necessary to block the descending aorta in an ischemia model of the spinal cord (Marsala et al., 1994). Lang-Lazdunski et al. (2000) established a model of spinal cord ischemia in mice by reperfusing the aortic arch and the left subclavicular artery after cross-foraging for different time periods followed by assessment of motor function scores and HE. A reproducible mouse ischemia model has been generated by cross-clipping of the aortic arch and the left subclavian artery. Compared to rats, mouse models have obvious advantages in genomic research.

## Pathological changes in the immune microenvironment in spinal cord injury

**FIGURE 3:**
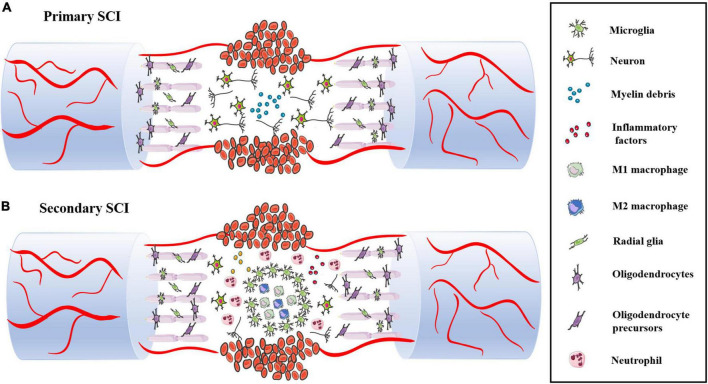
**(A)** The structure and content of spinal cord are destroyed during primary SCI, including neuron body and axon damage and destruction of the BSCB. **(B)** Following SCI, immune cells infiltrate into the damaged spinal cord and release various pro-inflammatory factors, which leads to massive neuronal death.

Primary SCI is a direct injury that shows destruction of spinal cord structure and contents caused by traction, shearing and compression from external forces. A series of complex and interrelated activities occurs at the site of injury, including the oxidative stress response, inflammatory response, delayed apoptosis and glial scar formation, which cause secondary SCI. In these activities, immune cells, such as monocytes, macrophages, neutrophils and microglia, play various roles, and they also interact with oligodendrocytes and astrocytes to guide the transformation between proinflammatory and anti-inflammatory cells in the immune microenvironment. Secondary SCI can be divided into acute, subacute and chronic stages according to the time course (Figure 3).

### Acute phase

The acute phase usually occurs within 48 h of SCI. The spinal cord structure is severely damaged after being impacted by external forces. The main pathological manifestations are destruction of the BSCB, severe bleeding, sever edema, ion imbalance, increased excitatory toxicity, free radical production and inflammatory reactions caused by activation and infiltration of immune cells.

#### Severe bleeding and ischemic edema

The blood–spinal cord barrier (BSCB) is composed of astrocytes, microglia surrounding blood vessels and continuous capillary endothelial cells in the basement membrane. These cells insulate and protect the spinal cord from metabolites and harmful substances in the blood and surrounding environment. Damage to the BSCB by mechanical external forces is one of the earliest events in the acute stage of SCI. Bleeding usually occurs in the central part of the spinal cord, occupying a major part of the gray matter and radiating into the white matter (Bartanusz et al., 2011). There is evidence that bleeding causes nerve tissue damage (Noble and Wrathall, 1989). After sustained bleeding, vascular ischemia, hypovolemia, and hyperfusion occur at the site of injury. Various phagocytes are activated and migrate to the site of injury and catalyze the release of metal ions, leading to the formation of free radicals. The nerve of the spinal cord is rich in fatty acid chains, which are sensitive to free radicals, ultimately leading to a large amount of neuronal cell death and tissue destruction (LeBel and Bondy, 1991). At the same time, the blood vessel ruptures, causing a large amount of immune cells (neutrophils, macrophages, microglia and lymphocytes) to extravasate into the blood. These immune cells migrate and ingest myelin fragments by sensing the injury signal. However, these immune cells impair the tight junctions and interstitial junctions of endothelial cells, leading to increased vascular permeability and leakage. Plasma-derived macromolecules penetrate cell membranes and cause edema.

#### Excitatory toxicity and ion imbalance

Excitatory toxicity and ion imbalance are markers in the acute stage of secondary SCI. Glutamate receptors are involved in the excitatory transmission of the central nervous system and are associated with various chemical changes in synaptic transmission. In primary SCI, neurons and glial cells release a large amount of glutamate, which overactivates ionic glutamate receptors. Calcium and sodium ion influx leads to cell necrosis, apoptosis or autophagy. In addition, the release of ATP and nucleotides activates purinergic receptors and leads to calcium influx.

#### Inflammatory response

The inflammatory response occurs throughout the process of SCI. Multiple immune cells are activated to rapidly migrate and infiltrate into the damaged spinal cord. Astrocytes, dendritic cells and other cells are also involved in the inflammatory response (Yang et al., 2018; Xing et al., 2019). In the first stage, neutrophils, resident microglia and astrocytes migrate to the site of injury, and they play a dual role in damage and repair during spinal cord regeneration. In the second stage, blood-derived macrophages and T/B lymphocytes produce various cytokines (IL-1α, IL-1β, IL-6 and TNF-α) and aggravate the inflammatory and oxidative reactions of the immune microenvironment. In this review, we will discuss the details in the next chapter. It is important to note that inflammation is more apparent in the acute phase of injury but still continues in subacute and chronic inflammation.

### Subacute stage

The subacute phase typically occurs within 48 h to two weeks after SCI. Neuron and oligodendrocyte apoptosis as well as axon demyelination occur in this stage. The appearance of glial scars around the lesion site and the release of axon growth inhibition factor are key events affecting spinal cord regeneration.

#### Apoptosis and axonal demyelination

Demyelination mainly occurs 2-7 days after SCI, and it initially occurs in the lesion center and then gradually moves to the fiber bundles of spinal white matter (Blight, 1985). Totoiu and Keirstead (2005) conducted a study on chronic pathological processes and found that the number of demyelinated axons peaks within one day of injury and then declines within 1-2 weeks with a process lasting up to 450 days. Therefore, it can be inferred that the pathological process of demyelination mainly occurs within two weeks, but this process may last for a long time. The process of demyelination is often accompanied by the apoptosis and necrosis of oligodendrocytes, which may be one of the important causes (Wu and Ren, 2008). A series of studies have demonstrated this view. Sanchez et al. (1996) showed that locally induced aggregation of neural fiber networks by oligodendrocytes is critical for axon growth. Traka et al. (2016) found that oligodendrocyte death results in extensive myelin and axon loss. Moore et al. (2015) showed that oligodendrocyte progenitor cells at the site of injury are affected by proinflammatory T cells, M1-polarized BM cells and microglia, resulting in reduced differentiation. In addition, other research on oligodendrocyte apoptosis has been reported, including excessive glutamate release, insufficient nutritional factors and activated caspases (Xu et al., 2004). Studies on spinal cord regeneration mainly involve inhibition of demyelination or promotion of myelin regeneration. Armstrong et al. (2002) found that an increase in FGF2 facilitates the proliferation of oligodendrocyte progenitor cells and reduces the number of differentiated mature glial cells (Armstrong et al., 2002). Schwann cells are generally considered to be myelinated cells of the peripheral nervous system, but some studies have shown that Schwann cell remyelination also occurs after central nervous damage. Franklin et al. reported that the Schwann cells in the central system migrate from peripheral nerves after the destruction of glial cells (Franklin and Blakemore, 1993). Keirstead et al. (1999) suggested that Schwann cell remyelination is due to the abnormal differentiation of endogenous precursor cells.

#### Glial scar formation and axon growth inhibition factor

As immune cells infiltrate, glial cells become activated, proliferate and eventually form glial scars, which blocks the inflammatory response caused by immune cell infiltration. However, scarring largely prevents neuron regeneration and differentiation. The mechanical barrier of axonal regeneration includes a variety of cells (astrocytes, oligodendrocyte progenitor cells, immune cells fibroblasts) and cell matrix compositions (Bradbury and Burnside, 2019; Lukacova et al., 2021). Activated astrocytes also have two different phenotypes, namely, A1 and A2, similar to macrophages. A1 astrocytes have neurotoxic effects and induce rapid neuronal and oligodendrocyte death, while A2 astrocytes have neuroprotective effects and promote neuronal survival (Wang et al., 2021).

In addition, the injured site microenvironment is a key factor. Growth-related inhibitory factors in the extracellular matrix, including chondroitin sulfate proteoglycan (CSPG), neurite outgrowth inhibitor (NOGO-A), myelin-associated glycoprotein (MAG) and oligodendrocyte myelin glycoprotein (OMgp), inhibit regeneration progression. CSPG is the major component of the glial scar after SCI. CSPG inhibits axon regeneration and plays a leading role in demyelination and axonal degeneration. Removal of glycosaminoglycan chains of CSPG attenuates the inhibitory activity in spinal cord regeneration. The traditional view is that astrocyte scar is not conducive to axon regeneration and its formation is one of the important reasons for the failure of axons regeneration. Anderson et al. (2016) proposed the opposite opinion. They demonstrated that astrocyte glial scarring contributes to rather than prevents axon regeneration in the central nervous system through loss of function. In addition, astrocyte scar formation may play a beneficial role in limiting inflammatory cell infiltration, filling the injured site, rebuilding the BCSC, and protecting neurons and oligodendrocytes (Bush et al., 1999; Sofroniew, 2005, 2015; Kawano et al., 2012; Bradbury and Burnside, 2019). The role of glial scar formation in recovery from spinal cord injury remains controversial.

### Chronic phase

In the chronic stage, the glial scar thickens, and regeneration becomes more difficult. In the acute stage, astrocyte scarring alleviates the inflammatory response at the site of SCI, however, it obstructs the regeneration and differentiation of neuron cells to a large extent (Anderson et al., 2016). Li et al. (2018b) reported that glial scars in subacute and chronic stages have different compositions, characteristics and effects. The treatment strategies for glial scars in different stages should also be different. Li et al. (2018b) also suggested that the glial scar has an inhibitory effect on axon regeneration and that removal of the glial scar in the chronic stage significantly increases axon regeneration. Some researchers have attempted to transplant stem cells at the chronic stage. Wertheim et al. removed the scar of the chronic stage and transplanted hydrogel-encapsulated iPSCs to the injured site, resulting in good functional recovery, and they also transplanted neural stem cells/progenitor cells from human olfactory epithelial mucosa, resulting in improved hind limb motor function of rats (Voronova et al., 2020).

At present, few studies have focused on the immune microenvironment of chronic stages after SCI. Beck et al. detected changes in major immune cell types at the early stage (10 days) and later stage (180 days) after SCI with immune cells still detected at 180 days after injury, and they reported that the inflammatory response has a reparative effect (Beck et al., 2010). These findings suggest SCI is a lengthy process, and the associated reactions may persist throughout the patient’s life. Dulin et al. reported increased levels of oxidative and inflammatory metabolites in the injured site of rats 9 months after SCI, and they demonstrated that licofelone reduces the expression level in the injured site (Dulin et al., 2013). Hains et al. intrathecally injected the microglial inhibitor, minocycline, in a rat contusion model and showed that activated microglia promote chronic central nerve pain.

## Immune cells in the microenvironment of spinal cord injury

**FIGURE 4:**
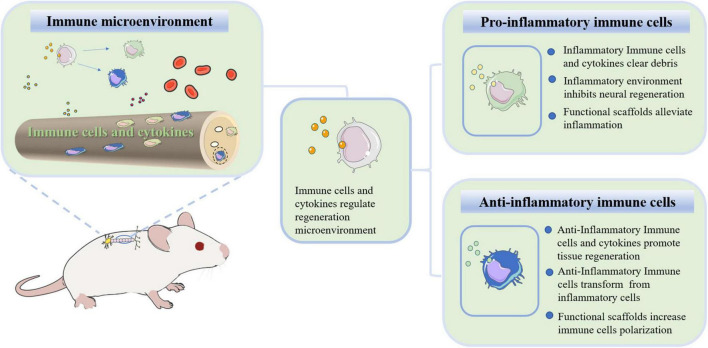
Changes of immune cells in the microenvironment of spinal cord injury.

Various inflammatory immune cells, such as neutrophils, monocytes and microglia, are activated by cytokines or chemokines, and they rapidly infiltrate through damaged blood vessels and participate in the activities of the immune microenvironment after SCI (Anwar et al., 2016; Yang et al., 2021c). Astrocytes, microglia and oligodendrocyte progenitors subsequently proliferate. Monocytes differentiate into macrophages and engulf damaged tissue or cell debris, and fibroblasts are stimulated and activated to induce fibrosis (Kong and Gao, 2017). To prevent further expansion of the damaged tissue, the fibrotic scar is gradually surrounded by an astrocyte scar, reaching a stable state after injury, which greatly obstructs the regeneration and differentiation of nerve cells (Figure 4).

### Neutrophils

Neutrophils are derived from BM hematopoietic stem cells and are abundant in the peripheral blood. Neutrophils are short-lived (∼24 h) innate immune cells, and they are removed by macrophages. Neutrophils are the first activated inflammatory cells that migrate to the injured spinal cord. Neutrophils cause oxidative and inflammatory responses, but the exact mechanism is still unclear. In addition, the degranulation and phagocytosis of neutrophils against myelin sheath fragments after SCI have become a new research direction.

#### Neutrophil activation and migration

The loss of injured nerve function is associated with neutrophil recruitment and accumulation at the local lesion site. Neutrophil receptors recognize two types of signaling, namely, pathogen-associated molecular patterns (PAMPs) and danger-associated molecular patterns (DAMPs), in response to pathogen invasion or tissue damage. After SCI, injured cells are damaged and release various DAMPs, including DNA, RNA, histones, high mobility group protein 1 (HMGB1) and adenosine triphosphate (ATP). The receptors that sense these signals activate serine-activated protein kinase and NF-κB downstream pathways to express more proinflammatory factors, thereby recruiting more neutrophils (Albrecht et al., 2007). Previous research has found that the expression of CCL2, CXCL1 and CXCL2 in astrocytes from 3 h to 12 h leads to neutrophil infiltration, which is related to the MyD88 and IL-1 receptor signaling pathways (Pineau et al., 2010). Other inflammatory, factors and chemokines included IL-1α, IL-1β, TNF, GCSF, CCL3, CXCL1, CXCL2, and CXCL5, are involved in this process (Zivkovic et al., 2021). Spleen tyrosine kinase (Syk) facilitates specific neutrophil functional responses to SCI, including activation, cytokine expression and cell death. Long-term neurological deficits are exacerbated by Syk signaling in neutrophils independent of acute blood–spinal cord barrier disruption and long-term white matter sparing (McCreedy et al., 2021). Generally, neutrophils begin to infiltrate the injured site within a few hours and peak within three days. Neutrophils produce more inflammatory factors, proteolytic enzymes, ROS and nitrogen substances, which deteriorate the microenvironment of the injured site, resulting in a large amount of cell apoptosis and necrosis (Okada, 2016).

#### Infiltrating neutrophils induce tissue damage

The adverse effects of neutrophil infiltration in the acute stage of SCI are inflammation, oxidative stress response, proinflammatory factors, hydrolase and other damage to the BSCB, which result in inadequate vascular formation. Neutrophils release harmful substances that are unfavorable to the injury microenvironment. These unfavorable factors include neutrophil matrix traps (NETs), neutrophil elastase (NE), myeloperoxidase (MPO) and metal matrix peptide enzymes (MMPs) (Fiani et al., 2021).

**FIGURE 5:**
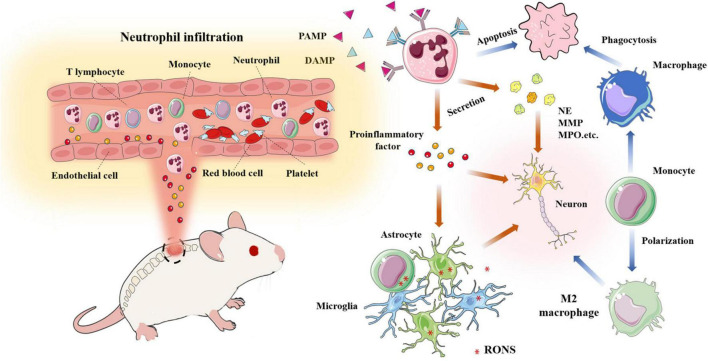
A large number of neutrophils infiltrate into the damaged site and release pro-inflammatory factors and chemokines; Neutrophils release various harmful enzymes (MMP, NE, MPO,etc); Activated monocytes, microglia, astrocytes,etc produce RONS, resulting in the deterioration of the microenvironment; Macrophages phagocytize apoptotic neutrophils and transform into M2 macrophages.

Neutrophil matrix traps are composed of chromatin fibers and granular proteins, including histones, granulocyte enzymes and peptides. Previous research has shown that NETs induce inflammatory responses and damage the BSCB, resulting in axonal degeneration, which is not conducive to neuronal regeneration. The arginine deaminase-4 peptide blocks the formation of NETs by DNAse-1, which reduces cell death and scar formation, thereby promoting functional recovery (Feng et al., 2021). In addition, NETs accelerate endothelial cell injury and destroy the BSCB by increasing the expression of TRPV4. Tonai et al. (2001) used the specific neutrophil protease inhibitor, ONO-5046, to significantly reduce nerve injury in a rat model of compression. MMP is an important extracellular matrix hydrolase that degrades basement membrane components. MMPs damage the BSCB and aggravate the microenvironment. MMPs participating in SCI mainly include MMP-1, MMP-2, MMP-9 and MMP-12. MMP-9 and MMP-2 are closely related to the loss of nerve function and formation of glial scars (Zhang et al., 2011). The expression of MMP-9 peaks at 12-24 h, which exacerbates secondary SCI (de Castro et al., 2000). In addition, MMP-9 restricts angiogenesis in the early stage of tissue damage (Figure 5; Noble et al., 2002).

After spinal cord injury, a large number of neutrophils with blood and other immune cells infiltrate the damaged site and release a large number of pro-inflammatory and chemotactic factors; Neutrophils release cytokines and various harmful enzymes (MMP, NE, mpo.,etc.); Various inflammatory mediators activate monocytes, microglia, astrocytes, etc. to produce reactive oxygen and nitrogen species, resulting in the deterioration of the microenvironment; Macrophages phagocytized apoptotic neutrophils and transformed into M2 macrophages.

#### Potential regenerative effects of neutrophils

Although most research has focused on the deleterious effects of neutrophils on SCI, neutrophils are heterogeneous, and the recruitment of subsets may not necessarily indicate an exacerbation of injury. Sas et al. (2020) discovered that a new subpopulation of neutrophils (CD14^+^ Ly6G*^low^*) promotes neuronal survival and axon regeneration in the central nervous system. Another study has shown that neutrophils activate more immune cells to migrate to the injury site and clear debris by secreting proinflammatory factors. Excessive infiltrating neutrophils are removed by macrophages, which induces their transformation into the M2 anti-inflammatory phenotype and promotes injury repair (Soehnlein and Lindbom, 2010). It has also been demonstrated that neutrophils play a role in inflammation and tissue repair to some extent by releasing secretory leukocyte proteases (Ghasemlou et al., 2010).

#### Engineered neutrophils promote spinal cord regeneration

Neutrophils activate and migrate to the injured site and release various proinflammatory and oxidative factors. Some researchers have applied these characteristics to loads with drugs or cytokines to improve the immune regeneration microenvironment. Tetramethylpyrazine (TMP) has anti-inflammatory, antioxidative, and neuroprotective effects, but it is not easily soluble in water. Li et al. (2021) developed nanoparticles that can be internalized by neutrophils. These nanoparticles rapidly infiltrate into the injury site of the spinal cord in the acute phase, and the drug-loaded nanoparticles significantly improve the immune regeneration microenvironment (Li et al., 2021). Yihui et al. developed a neutrophil membrane-encapsulated polydopamine nanoparticle, which effectively adsorbs various inflammatory factors and inhibits the production of ROS and nitrogen substances. The improved local immune microenvironment promotes neuronal growth, differentiation and motor recovery in rats (Bi et al., 2021).

### Macrophages

**FIGURE 6:**
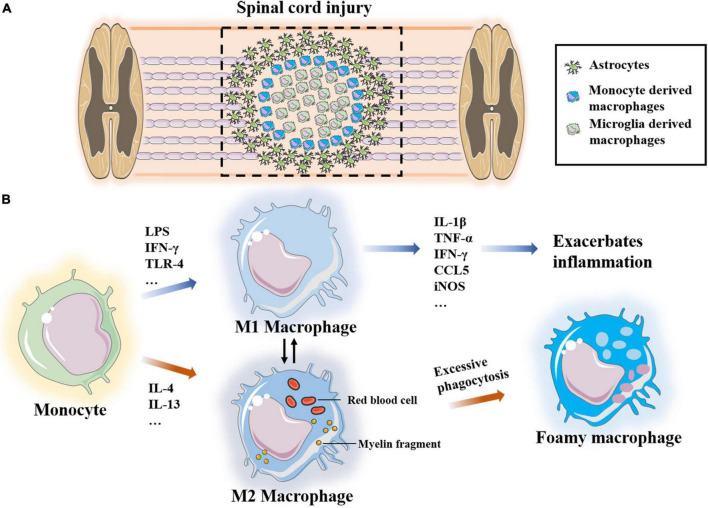
**(A)** Microglia-derived macrophages form boundaries at the site of injury and monocyte-derived macrophages infiltrate into the site of injury. **(B)** Monocytes polarize into two different phenotypes M1 and M2 under the stimulation of different factors, which transform into each other. Excessive phagocytosis of myelin sheath fragments and apoptotic cells induce macrophages transform into foam macrophages.

At the central site of SCI, macrophages are heterogeneous and mostly derived from BM. Macrophages are responsible for removing debris from damaged cells and tissues, while specialized activated microglia (central nerve resident cells) form boundaries at the damaged site (David and Kroner, 2011). Two sources of macrophages are indistinguishable in tissue sections and are termed microglia/macrophages after staining of the CD11b and IBA-1 markers. Considering some similar features, monocyte-derived macrophages and neuroresident microglia have been discussed together in many studies. However, these cells vary widely in terms of origin, biomarkers and function. After tissue damage, microglia recognize the injury signal from the surrounding environment and thus activate and infiltrate to the injury site following monocyte-derived macrophages (MDMs) (Ding et al., 2021; Sun et al., 2021). In addition, microglia contain fragments of phagocytic tissue 3 days after SCI, which is before MDM migration (David et al., 2018). Microglia and macrophages may have different roles in the injury microenvironment. In the next section, we discuss microglia and macrophages from two different sources. We first introduce monocyte-derived macrophages that migrate to the lesion site after activation from BM or the spleen (Figure 6).

#### Recruitment and polarization of monocyte-derived macrophages

Under the condition of injury, specific cytokines stimulate macrophages to differentiate into different phenotypes, namely, pro-inflammatory M1 and anti-inflammatory M2 macrophages (Kroner and Rosas Almanza, 2019). M1 macrophages are neurotoxic and inflammatory, and they are induced by LPS, IFN-γ and Th1 cytokines. M1 macrophages also produce many inflammatory cytokines and chemokines, such as IL-1β, IL-6, IL-12, IL-23, TNF-α, IFN-γ, CCL5, and iNOS, as well as proteolytic enzymes to clean up myelin sheath fragments and apoptotic cells as well as to promote tissue remodeling. Previous research has shown that chondroitin sulfate proteoglycan is 17 times higher in M1 macrophages than in M2 macrophages. Chondroitin sulfate proteoglycan is an inhibitor of axon growth, suggesting that M1 macrophages are harmful to axons (Tan et al., 2005; Martinez et al., 2006). In addition, M1-derived IL-6 disrupts the tight junctions of endothelial cells, increases vascular permeability and promotes continuous leakage of the BSCB. The consumption of macrophages significantly improves vascular-to-barrier leakage and promotes the functional recovery of SCI (Luo et al., 2022). Ge et al. (2021) also confirmed that miR-155 from M1-polarized macrophage exosomes impairs the mitochondrial function of vascular endothelial cells and destroys the BSCB in the SCI microenvironment. However, these results do not indicate that M1 macrophages have no role in the repair and regeneration of SCI. M1 macrophages phagocytose and clear damaged tissues and apoptotic cells, and they promote axon growth to a certain extent (Kong and Gao, 2017; Kroner and Rosas Almanza, 2019).

Monocytes polarize to M2 macrophages under the stimulation of the Th2 cytokines, IL-4 and IL-13. There are three subpopulations of M2 macrophages, namely, M2a, M2b and M2c. M2a macrophages are mainly involved in reducing inflammation and enhancing phagocytosis and differentiation of neural stem cells. M2b macrophages produce CCL1, IL-10, ROS and nitrogen substances during the activation of M1 macrophages cells. M2c macrophages clear myelin sheath debris and promote healing (Kong and Gao, 2017). Most studies have suggested that M2-type macrophages promote the regeneration of the injury microenvironment. After adoptive transfer of M2 macrophages into a rat SCI model, Ma et al. (2015) reported that the secreted IL-10 and TGF-β induce polarization of microglia/macrophages and significantly improve the recovery of neurological function. Peng et al. (2021) applied M2-derived exosomes to reduce the proportion of M1 macrophages and increase the proportion of M2 cells, and they found that M2 polarization mainly occurred through the miRNA–mRNA network and that the miR-23a-3p/PTEN/PI3K/AKT axis plays an important role in this process. Yao et al. (2014) demonstrated that PD-1 may play an important role in macrophage polarization, and they reported that increased expression of transcriptional activator-1 in M1 macrophages of PD-1 knockout mice promotes M2 transformation.

#### Phagocytosis of monocyte-derived macrophages

Macrophages induce a dual role of inflammation and phagocytosis. On the one hand, the removal of myelin sheath fragments and apoptotic cells is conducive to improving the microenvironment of the injury site; on the other hand, inflammation, chemokines, ROS and excessive phagocytosis lead to the formation of harmful foam macrophages. After phagocytosis of myelin after nerve injury, macrophages are filled with lipid droplets and transform into foam-like cells, which lose the ability to clean up residual debris. Kong et al. (2020) reported that scavenger receptor (SR) promotes excessive phagocytosis fragments in macrophages, resulting in foam-like macrophage-induced inflammation (Yao et al., 2014; Kong et al., 2020). Kroner et al., 2014 showed that macrophages accumulate a large amount of iron after phagocytosis and induce the expression of TNF, which contributes to the maintenance of the M2-induced regeneration microenvironment.

#### Biomaterials regulate macrophages and improve the immune microenvironment

Shen et al. (2022b) optimized photocrosslinked gelatin hydrogels with polyamine-amine dendritic macromolecules (PAMAM-G3) and IL-10, and they demonstrated that the hydrogels inhibit the inflammatory response of monocytes/macrophages, regulate M2 polarization and promote the differentiation and regeneration of neuronal cells. Jeong et al. (2017) injected PLGA-modified nanoparticles with the macrophage receptor and collagenous structure; these nanoparticles not only significantly reduce macrophage polarization and glial scar formation but also reduce the accumulation of chondroitin sulfate proteoglycan, and the regeneration of axons promotes motor function recovery. Bartus et al. (2014) applied a lentivirus vector to deliver the chondroitin sulfate gene to regulate the phenotype of macrophages and protect injured nerves, and they reported that the polarized M2 macrophages reduce the number of glial cells (astrocytes and microglia) and promote the immune regeneration microenvironment through the expression of TNF-β and IL-10. Kigerl et al. (2009) reported that M2 macrophage-conditioned medium promotes long and extensive neurites of dorsal root ganglion neurons, and they also demonstrated that M1 macrophage-conditioned medium induces short and stunted neurites with multiple branches. Li X. et al., 2020 developed nanofiber hydrogels using maleimide-modified PCL fibers connected to hyaluronic acid, and they reported that these hydrogels induce M2 polarization and significantly reduce inflammation and vascularization as well as promote neuronal cell regeneration and differentiation after SCI.

### Microglia

Microglia are derived from the embryonic yolk sac, and they are differentiated from border macrophages distributed around the vascular space outside the spinal cord parenchyma. As innate resident immune cells, microglia rapidly change their morphology after being stimulated by environmental signals, such as hypoxia or injury. Microglia express high levels of CD86 and MHCII, and they secrete a variety of proinflammatory or chemokines to promote immune cell infiltration. One day after spinal cord contusion, approximately 33% of microglia migrate to the injury site, and this number reaches half of all immune cells within 4 days. Furthermore, the microglia began to release CD68, and the number peaks at 7 days. The phagocytic capacity of microglia decreases after 14 days (Xu et al., 2021). Guo et al. (2013) reported that G-CSF improves the inflammatory response in the microglial environment and increases the expression of trophic factors. Another study has reported that SCI can heal without forming glial scars in newborn mice, in which microglia play an important role (Li Y. et al., 2020).

### T lymphocytes

The effect of T cells after nerve injury is often contradictory, which may be due to T cells differentiating into various subpopulations. According to phenotype, T cells are divided into CD4^+^ T cells and CD8^+^ T cells (Yang et al., 2021b). Furthermore, the subpopulations of CD4^+^ T cells include helper T cells (Th1, Th2 and Th17) and regulatory T cells (Tregs) (Yang et al., 2019; Hu et al., 2021). Both CD4^+^ T and CD8^+^ T cells migrate to the injured site of the central nervous system to induce inflammation and neurodegeneration. Th1 cells activate macrophages by secreting IL-2 and IFN-γ, and Th2 cells mainly produce IL-4, IL-5, and IL-13. In addition, Tregs secrete high levels of IL-10 and TGF-β to modulate adaptive immune responses. At present, there are few studies on the role of T cells in nerve regeneration and protection. One study has shown that Th1 and Th2 both play protective roles in the central nervous system. Hu et al. (2016) adoptively transferred myelin basic protein to activate Th1 cells and induce higher levels of proinflammatory cells and cytokines in a rat model. In contrast, myelin basic protein-activated Th2 cells are more beneficial to the recovery of motor function in rats through anti-inflammatory cytokines. Studies have also shown that Th2 cells secrete more neurotrophic factors than Th1 and Th17 cells in CNS injury (Hendrix and Nitsch, 2007). Other research has found that Th1 cells are more conducive to regeneration. Ishii et al. (2012) showed that proinflammatory Th1 cells secrete IFN-γ and IL-10 to promote functional recovery after SCI. The more pronounced neuroprotective effect of Th1 cells has been verified in a mouse model of SCI (Ishii et al., 2012). T cells mostly indirectly regulate the regeneration and repair of injured nerves, and few reports have directly demonstrated their effects. One study has shown that Tregs promote the differentiation of oligodendrocyte progenitor cells into mature oligodendrocytes after central nervous system injury, indicating that T cells directly regulate injury nerve regeneration (Dombrowski et al., 2017).

## Conclusion and perspectives

Immune cells play an important role in the recognition of SCI, remodeling of the microenvironment and tissue regeneration. Few studies have discussed the immune microenvironment after SCI. In this review, different pathological characteristics and changes in immune cells after SCI were described in detail. Neutrophils, macrophages, microglia and T lymphocytes play a dual role in injury and repair. On the one hand, these cells eliminate adverse factors and promote regeneration. Monocytes and microglia migrate to the damaged site to phagocytose and remove the damaged tissue debris, which is beneficial to the microenvironment. Th2 cells secrete IL-4 and IL-13, mediating M2 macrophage-promoted axon regeneration. The neutrophil subsets and Tregs have the ability to repair injured tissue. On the other hand, the remodeling of the microenvironment caused by immune cells may be highly inflammatory. Immune cell infiltration tends to release a large number of factors that induce inflammation and oxidative responses. Excessive phagocytosis results in the formation of foamy macrophages. Th1 cells release IFN-γ and IL-6 to activate macrophages, which have a proinflammatory role, thereby disrupting the balance of the immune system.

Functional nanoparticles, hydrogels and acellular matrix scaffolds have been widely used in tissue engineering regeneration and repair. Optimizing the physical and chemical properties of biomaterials has been explored. Biomaterial scaffold-loaded immune cells or factors slow inflammatory reactions and rebalance the regenerative microenvironment. There are three ways to regulate the immune response with biomaterials. First, the regulated immune biomaterial itself inhibits the inflammatory response of the microenvironment. Second, biomaterials loaded with inhibitors or anti-inflammatory factors promote nerve regeneration. Third, biomaterials encapsulate stem cells or immune cells that can interact with the immune microenvironment. These strategies effectively improve the microenvironment after SCI and promote the occurrence of new neurons. With the help of various biomaterials, the immunological principles described above show great potential. However, it should be noted that biomaterials loading a single cell or factor cannot solve all the problems in such a complex and spatiotemporal dynamic microenvironment at present. The combination of multiple factors and a precise engineering strategy may provide a promising future.

## Author contributions

YF: conceptualization and writing – original draft. YP: writing – original draft. JJ: writing – review editing, project administration, and funding acquisition. YY: supervision. PY: conceptualization, writing – original draft, review and editing, supervision, project administration, and funding acquisition. All authors contributed to the article and approved the submitted version.
